# Ethyl 4-(3-butyrylthio­ureido)benzoate

**DOI:** 10.1107/S1600536808017868

**Published:** 2008-06-28

**Authors:** Sohail Saeed, Moazzam Hussain Bhatti, Muhammad Kalim Tahir, Peter G. Jones

**Affiliations:** a Department of Chemistry, Allama Iqbal Open University, Islamabad, Pakistan; b Institut für Anorganische und Analytische Chemie, Technische Universität Braunschweig, Postfach 3329, 38023 Braunschweig, Germany

## Abstract

The title compound, C_14_H_18_N_2_O_3_S, crystallizes in the thio­amide form with an intra­molecular N—H⋯O hydrogen bond associated with the thio­urea unit. With the benzoic acid and the butyrylthio­ureido units, the mol­ecule consists of two planar building blocks connected by the common NH function adjacent to the aromatic ring. The inter­planar angle is 33.38 (3)°. Mol­ecules are connected in chains parallel to [110] by classical hydrogen bonds of the N—H⋯O type from the other NH group to the benzoate C=O of a neighboring mol­ecule.

## Related literature

For related literature, see: del Campo *et al.* (2002 ▶); D’hooghe *et al.* (2005 ▶); Dušek (1985 ▶); Huebner *et al.* (1953 ▶); Rodriguez-Fernandez *et al.* (2005 ▶); Xu *et al.* (2004 ▶); Zeng *et al.* (2003 ▶).
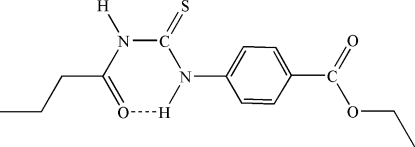


## Experimental

### 

#### Crystal data

C_14_H_18_N_2_O_3_S
*M* *_r_* = 294.36
Triclinic, 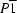

*a* = 7.9817 (4) Å
*b* = 9.8843 (6) Å
*c* = 11.0759 (6) Å
α = 114.472 (6)°
β = 101.156 (4)°
γ = 102.277 (5)°
*V* = 737.15 (7) Å^3^
*Z* = 2
Mo *K*α radiation
μ = 0.23 mm^−1^
*T* = 100 (2) K
0.28 × 0.18 × 0.12 mm

#### Data collection

Oxford Diffraction Xcalibur S diffractometer
Absorption correction: multi-scan (*CrysAlis RED*; Oxford Diffraction, 2008 ▶) *T*_min_ = 0.944, *T*_max_ = 1.000 (expected range = 0.918–0.973)
15025 measured reflections
4104 independent reflections
3045 reflections with *I* > 2σ(*I*)
*R*_int_ = 0.036

#### Refinement

*R*[*F* ^2^ > 2σ(*F* ^2^)] = 0.037
*wR*(*F* ^2^) = 0.096
*S* = 0.96
4104 reflections
191 parameters
1 restraint
H atoms treated by a mixture of independent and constrained refinement
Δρ_max_ = 0.45 e Å^−3^
Δρ_min_ = −0.24 e Å^−3^

### 

Data collection: *CrysAlis CCD* (Oxford Diffraction, 2008 ▶); cell refinement: *CrysAlis CCD*; data reduction: *CrysAlis RED* (Oxford Diffraction, 2008 ▶); program(s) used to solve structure: *SHELXS97* (Sheldrick, 2008 ▶); program(s) used to refine structure: *SHELXL97* (Sheldrick, 2008 ▶); molecular graphics: *XP* (Siemens, 1994 ▶); software used to prepare material for publication: *SHELXL97*.

## Supplementary Material

Crystal structure: contains datablocks I, global. DOI: 10.1107/S1600536808017868/im2072sup1.cif

Structure factors: contains datablocks I. DOI: 10.1107/S1600536808017868/im2072Isup2.hkl

Additional supplementary materials:  crystallographic information; 3D view; checkCIF report

## Figures and Tables

**Table 1:** Hydrogen-bond geometry (Å, °)

*D*—H⋯*A*	*D*—H	H⋯*A*	*D*⋯*A*	*D*—H⋯*A*
N2—H02⋯O1	0.82 (2)	1.92 (2)	2.653 (1)	148 (2)
N1—H01⋯O2^i^	0.79 (1)	2.20 (1)	2.957 (1)	160 (2)
C13—H13*A*⋯O1^ii^	0.99	2.58	3.363 (2)	136
C1—H1*B*⋯S^iii^	0.98	3.00	3.854 (2)	147
C13—H13*B*⋯S^iv^	0.99	2.96	3.577 (1)	122
C14—H14*C*⋯S^v^	0.98	2.98	3.821 (2)	144

Symmetry codes: (i) 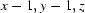; (ii) 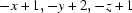; (iii) 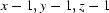; (iv) 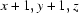; (v) 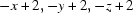.

**Fig. 1.**
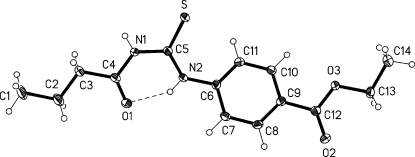
The molecular structure of the title compound in the crystal. Ellipsoids represent 50% probability levels.

**Fig. 2.**
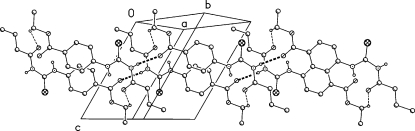
Packing diagram of I showing classical and "weak" H bonds as thick or thin dashed bonds respectively. The double chain pattern is apparent.

## supplementary crystallographic information

### Comment

Epoxy resins have the combination of good thermal and dimensional stability, excellent chemical and corrosion resistance, high tensile strength and modulus, and ease of handling and processability, ensuring their wide application in the aerospace and electronic industries in the form of structural adhesives, advanced composite matrices, and packaging materials (Dušek, 1985). The properties of cured epoxy polymers largely depend on the nature of chemical structure of the starting resins and curing agents. The title compound (I) is a precursor in an attempt to synthesize imidazole derivatives and transition metal complexes as epoxy resin curing agents and accelerators. Substituted thioureas are an important class of compounds, precursors or intermediates towards the synthesis of a variety of heterocyclic systems such as imidazole-2-thiones (Zeng *et al.*, 2003), 2-imino-1,3-thiazolines (D'hooghe *et al.*, 2005), pyrimidine-2-thiones and (benzothiazolyl)-4-quinazolinones. Thioureas are also known to exhibit a wide range of biological activities including antiviral, antibacterial, antifungal, antitubercular, antithyroidal, herbicidal and insecticidal activities (Huebner *et al.*, 1953) and as agrochemicals (Xu *et al.*, 2004). Among thiourea derivatives, acylthioureas, with O and S as potential donor sites, have been found to display a remarkably rich coordination chemistry. Such coordination compounds of thiourea have been studied for various biological systems (Rodriguez-Fernandez *et al.*, 2005). In recent years some attention has also been paid to the potential use of acylthioureas as highly selective reagents for the enrichment and separation of metal cations (del Campo *et al.*,2002).

The title compound crystallizes in the thioamide form with an intramolecular hydrogen bond N2—H02···O1. Bond lengths and angles (*cf.* Supplementary Material) may be regarded as normal. The molecule consists of two planar building blocks: the butyrylthioureido group (S, C1–5, O1, N1, N2) and the benzoic acid moiety (C6–14, N2, O2, O3). Mean deviations from planarity for these moieties are 0.12 and 0.13 Å, respectively, and the interplanar angle is 33.38 (3)°. Molecules are connected to give infinite chains parallel to [110] by classical hydrogen bonds N1—H01···O2. These are in turn connected to antiparallel chains by the weak hydrogen bonds C13—H13A···O1. Additionally, there are three C—H···S contacts that may be borderline weak H bonds (Table 1, Fig. 2).

### Experimental

A mixture of ammonium thiocyanate (26 mmol) and butanoyl chloride (26 mmol) in anhydrous acetone (70 ml) was stirred for 35 min. Then *p*-aminobenzoic acid ethyl ester (26 mmol) was added dropwise and the reaction mixture was refluxed for 2 h. After cooling, the reaction mixture was poured in acidified cold water. The resulting light green solid was filtered and washed with cold acetone.The product was recrystallized from ethanol as light greenish crystals (3.62 g, 91%), m.p. 412 K.

### Refinement

The NH H atoms were refined freely but with distance restraints (command SADI). Methyl H atoms were included on the basis of idealized rigid groups (C—H 0.98 Å, H—C—H 109.5°) allowed to rotate but not tip. Other hydrogen atoms were included using a riding model with C—H 0.95 (aromatic) or 0.99 (methylene) Å. U(H) values were fixed at 1.5*U*_iso_(C) of the parent C atom for methyl H, 1.2*U*_iso_(C) for other H.

### Crystal data

**Table:**

C_14_H_18_N_2_O_3_S	*Z* = 2
*M**_r_* = 294.36	*F*_000_ = 312
Triclinic, *P*1	*D*_x_ = 1.326 Mg m^−^^3^
Hall symbol: -P 1	Melting point: 412 K
*a* = 7.9817 (4) Å	Mo *K*α radiation λ = 0.71073 Å
*b* = 9.8843 (6) Å	Cell parameters from 7295 reflections
*c* = 11.0759 (6) Å	θ = 2.8–30.7º
α = 114.472 (6)º	µ = 0.23 mm^−^^1^
β = 101.156 (4)º	*T* = 100 (2) K
γ = 102.277 (5)º	Pyramid, colourless
*V* = 737.15 (7) Å^3^	0.28 × 0.18 × 0.12 mm

### Data collection

**Table:**

Oxford Diffraction Xcalibur S diffractometer	4104 independent reflections
Radiation source: Enhance (Mo) X-ray Source	3045 reflections with *I* > 2σ(*I*)
Monochromator: graphite	*R*_int_ = 0.036
Detector resolution: 16.1057 pixels mm^-1^	θ_max_ = 30.8º
*T* = 100(2) K	θ_min_ = 2.8º
ω scans	*h* = −10→11
Absorption correction: multi-scan(CrysAlis RED; Oxford Diffraction, 2008)	*k* = −14→13
*T*_min_ = 0.944, *T*_max_ = 1.000	*l* = −15→15
15025 measured reflections	

### Refinement

**Table:**

Refinement on *F*^2^	Secondary atom site location: difference Fourier map
Least-squares matrix: full	Hydrogen site location: inferred from neighbouring sites
*R*[*F*^2^ > 2σ(*F*^2^)] = 0.037	H atoms treated by a mixture of independent and constrained refinement
*wR*(*F*^2^) = 0.096	*w* = 1/[σ^2^(*F*_o_^2^) + (0.059*P*)^2^] where *P* = (*F*_o_^2^ + 2*F*_c_^2^)/3
*S* = 0.96	(Δ/σ)_max_ = 0.001
4104 reflections	Δρ_max_ = 0.45 e Å^−^^3^
191 parameters	Δρ_min_ = −0.24 e Å^−^^3^
1 restraint	Extinction correction: none
Primary atom site location: structure-invariant direct methods	

### Special details

**Table:**

Geometry. All e.s.d.'s (except the e.s.d. in the dihedral angle between two l.s. planes) are estimated using the full covariance matrix. The cell e.s.d.'s are taken into account individually in the estimation of e.s.d.'s in distances, angles and torsion angles; correlations between e.s.d.'s in cell parameters are only used when they are defined by crystal symmetry. An approximate (isotropic) treatment of cell e.s.d.'s is used for estimating e.s.d.'s involving l.s. planes.Least-squares planes (*x*,*y*,*z* in crystal coordinates) and deviations from them (* indicates atom used to define plane)- 7.3219 (0.0010) *x* + 3.9334 (0.0038) *y* + 4.1423 (0.0037) *z* = 2.1058 (0.0010)* -0.1410 (0.0006) S * -0.1556 (0.0012) C1 * -0.0747 (0.0014) C2 * 0.1521 (0.0012) C3 * 0.1191 (0.0012) C4 * -0.0053 (0.0010) C5 * 0.0111 (0.0008) O1 * 0.2033 (0.0010) N1 * -0.1089 (0.0009) N2Rms deviation of fitted atoms = 0.1249- 6.2227 (0.0013) *x* + 7.6804 (0.0015) *y* - 1.8402 (0.0041) *z* = 2.0195 (0.0017)Angle to previous plane (with approximate e.s.d.) = 33.38 (0.03)* -0.0510 (0.0011) C6 * -0.1492 (0.0011) C7 * -0.0777 (0.0011) C8 * 0.0751 (0.0012) C9 * 0.1972 (0.0012) C10 * 0.1468 (0.0011) C11 * 0.0732 (0.0011) C12 * 0.0367 (0.0013) C13 * -0.2621 (0.0011) C14 * 0.0788 (0.0009) O2 * 0.0421 (0.0010) O3 * -0.1099 (0.0009) N2Rms deviation of fitted atoms = 0.1266
Refinement. Refinement of *F*^2^ against ALL reflections. The weighted *R*-factor *wR* and goodness of fit *S* are based on *F*^2^, conventional *R*-factors *R* are based on *F*, with *F* set to zero for negative *F*^2^. The threshold expression of *F*^2^ > σ(*F*^2^) is used only for calculating *R*-factors(gt) *etc*. and is not relevant to the choice of reflections for refinement. *R*-factors based on *F*^2^ are statistically about twice as large as those based on *F*, and *R*- factors based on ALL data will be even larger.

### Fractional atomic coordinates and isotropic or equivalent isotropic displacement parameters (Å^2^)

**Table:**

	*x*	*y*	*z*	*U*_iso_*/*U*_eq_	
S	0.47787 (5)	0.62325 (4)	0.72720 (3)	0.02040 (10)	
O1	0.11708 (12)	0.45529 (11)	0.28568 (9)	0.0208 (2)	
O2	1.03683 (13)	1.25923 (10)	0.60929 (9)	0.0217 (2)	
O3	1.04639 (12)	1.31498 (10)	0.82958 (9)	0.0200 (2)	
N1	0.20683 (14)	0.45249 (13)	0.49337 (11)	0.0164 (2)	
H01	0.186 (2)	0.3993 (17)	0.5294 (16)	0.020 (4)*	
N2	0.38248 (15)	0.68105 (13)	0.51142 (11)	0.0168 (2)	
H02	0.318 (2)	0.6327 (19)	0.4292 (16)	0.034 (5)*	
C1	−0.2318 (2)	−0.03015 (17)	0.08970 (15)	0.0334 (4)	
H1A	−0.1512	−0.0873	0.1061	0.050*	
H1B	−0.2954	−0.0787	−0.0107	0.050*	
H1C	−0.3200	−0.0343	0.1396	0.050*	
C2	−0.1213 (2)	0.14034 (17)	0.14262 (14)	0.0313 (3)	
H2A	−0.0333	0.1442	0.0912	0.038*	
H2B	−0.2027	0.1971	0.1238	0.038*	
C3	−0.02144 (18)	0.22120 (15)	0.29715 (13)	0.0195 (3)	
H3A	−0.1110	0.2245	0.3480	0.023*	
H3B	0.0497	0.1574	0.3162	0.023*	
C4	0.10397 (16)	0.38601 (15)	0.35434 (13)	0.0161 (3)	
C5	0.35361 (16)	0.58945 (14)	0.57240 (12)	0.0152 (2)	
C6	0.52972 (17)	0.81999 (14)	0.56141 (13)	0.0158 (2)	
C7	0.60248 (17)	0.84283 (15)	0.46402 (13)	0.0177 (3)	
H7	0.5545	0.7659	0.3681	0.021*	
C8	0.74473 (18)	0.97773 (15)	0.50722 (13)	0.0174 (3)	
H8	0.7932	0.9937	0.4405	0.021*	
C9	0.81749 (17)	1.09026 (14)	0.64780 (13)	0.0162 (3)	
C10	0.74195 (17)	1.06811 (14)	0.74442 (13)	0.0176 (3)	
H10	0.7898	1.1453	0.8402	0.021*	
C11	0.59726 (18)	0.93406 (15)	0.70161 (13)	0.0183 (3)	
H11	0.5448	0.9203	0.7675	0.022*	
C12	0.97646 (17)	1.22913 (14)	0.69088 (13)	0.0168 (3)	
C13	1.20350 (18)	1.45409 (16)	0.88185 (14)	0.0237 (3)	
H13A	1.1674	1.5352	0.8632	0.028*	
H13B	1.2942	1.4265	0.8350	0.028*	
C14	1.28094 (19)	1.51477 (17)	1.03564 (14)	0.0267 (3)	
H14A	1.1882	1.5369	1.0802	0.040*	
H14B	1.3836	1.6116	1.0751	0.040*	
H14C	1.3218	1.4356	1.0525	0.040*	

### Atomic displacement parameters (Å^2^)

**Table:**

	*U*^11^	*U*^22^	*U*^33^	*U*^12^	*U*^13^	*U*^23^
S	0.02247 (18)	0.01856 (17)	0.01304 (16)	−0.00118 (13)	−0.00170 (12)	0.00807 (12)
O1	0.0212 (5)	0.0233 (5)	0.0159 (5)	0.0035 (4)	0.0012 (4)	0.0114 (4)
O2	0.0249 (5)	0.0201 (5)	0.0190 (5)	0.0014 (4)	0.0087 (4)	0.0103 (4)
O3	0.0195 (5)	0.0196 (5)	0.0147 (4)	−0.0025 (4)	0.0005 (4)	0.0091 (4)
N1	0.0176 (5)	0.0163 (5)	0.0118 (5)	0.0002 (4)	0.0020 (4)	0.0072 (4)
N2	0.0179 (5)	0.0166 (5)	0.0114 (5)	0.0007 (4)	0.0007 (4)	0.0069 (4)
C1	0.0376 (9)	0.0212 (7)	0.0205 (7)	0.0011 (6)	−0.0044 (6)	0.0010 (6)
C2	0.0431 (9)	0.0214 (7)	0.0159 (7)	0.0027 (6)	−0.0031 (6)	0.0059 (6)
C3	0.0197 (6)	0.0180 (6)	0.0138 (6)	0.0018 (5)	0.0023 (5)	0.0046 (5)
C4	0.0147 (6)	0.0193 (6)	0.0126 (6)	0.0058 (5)	0.0036 (5)	0.0062 (5)
C5	0.0153 (6)	0.0146 (6)	0.0138 (6)	0.0040 (5)	0.0046 (5)	0.0055 (5)
C6	0.0153 (6)	0.0149 (6)	0.0166 (6)	0.0032 (5)	0.0030 (5)	0.0085 (5)
C7	0.0218 (7)	0.0169 (6)	0.0128 (6)	0.0053 (5)	0.0041 (5)	0.0066 (5)
C8	0.0220 (6)	0.0178 (6)	0.0155 (6)	0.0063 (5)	0.0071 (5)	0.0102 (5)
C9	0.0174 (6)	0.0152 (6)	0.0169 (6)	0.0043 (5)	0.0045 (5)	0.0093 (5)
C10	0.0215 (6)	0.0159 (6)	0.0132 (6)	0.0043 (5)	0.0040 (5)	0.0065 (5)
C11	0.0215 (6)	0.0188 (6)	0.0157 (6)	0.0050 (5)	0.0073 (5)	0.0093 (5)
C12	0.0186 (6)	0.0165 (6)	0.0170 (6)	0.0063 (5)	0.0048 (5)	0.0096 (5)
C13	0.0192 (7)	0.0222 (7)	0.0232 (7)	−0.0037 (5)	−0.0011 (5)	0.0135 (6)
C14	0.0227 (7)	0.0281 (8)	0.0198 (7)	0.0003 (6)	0.0025 (5)	0.0086 (6)

### Geometric parameters (Å, °)

**Table:**

S—C5	1.6617 (13)	C13—C14	1.4939 (18)
O1—C4	1.2207 (15)	N1—H01	0.791 (13)
O2—C12	1.2114 (15)	N2—H02	0.823 (15)
O3—C12	1.3336 (15)	C1—H1A	0.9800
O3—C13	1.4576 (15)	C1—H1B	0.9800
N1—C5	1.3850 (16)	C1—H1C	0.9800
N1—C4	1.3856 (16)	C2—H2A	0.9900
N2—C5	1.3443 (16)	C2—H2B	0.9900
N2—C6	1.4161 (16)	C3—H3A	0.9900
C1—C2	1.520 (2)	C3—H3B	0.9900
C2—C3	1.5071 (18)	C7—H7	0.9500
C3—C4	1.5044 (17)	C8—H8	0.9500
C6—C11	1.3929 (17)	C10—H10	0.9500
C6—C7	1.3930 (17)	C11—H11	0.9500
C7—C8	1.3833 (17)	C13—H13A	0.9900
C8—C9	1.3926 (17)	C13—H13B	0.9900
C9—C10	1.3944 (17)	C14—H14A	0.9800
C9—C12	1.4846 (17)	C14—H14B	0.9800
C10—C11	1.3896 (17)	C14—H14C	0.9800
			
C12—O3—C13	116.02 (10)	C2—C1—H1C	109.5
C5—N1—C4	129.07 (11)	H1A—C1—H1C	109.5
C5—N2—C6	127.24 (11)	H1B—C1—H1C	109.5
C3—C2—C1	111.68 (12)	C3—C2—H2A	109.3
C4—C3—C2	114.61 (11)	C1—C2—H2A	109.3
O1—C4—N1	122.53 (12)	C3—C2—H2B	109.3
O1—C4—C3	123.91 (11)	C1—C2—H2B	109.3
N1—C4—C3	113.56 (11)	H2A—C2—H2B	107.9
N2—C5—N1	114.66 (11)	C4—C3—H3A	108.6
N2—C5—S	126.56 (9)	C2—C3—H3A	108.6
N1—C5—S	118.75 (9)	C4—C3—H3B	108.6
C11—C6—C7	120.20 (11)	C2—C3—H3B	108.6
C11—C6—N2	122.15 (11)	H3A—C3—H3B	107.6
C7—C6—N2	117.61 (11)	C8—C7—H7	120.1
C8—C7—C6	119.86 (11)	C6—C7—H7	120.1
C7—C8—C9	120.51 (11)	C7—C8—H8	119.7
C8—C9—C10	119.35 (12)	C9—C8—H8	119.7
C8—C9—C12	118.79 (11)	C11—C10—H10	119.7
C10—C9—C12	121.84 (11)	C9—C10—H10	119.7
C11—C10—C9	120.51 (12)	C10—C11—H11	120.2
C10—C11—C6	119.52 (11)	C6—C11—H11	120.2
O2—C12—O3	124.13 (12)	O3—C13—H13A	110.2
O2—C12—C9	123.78 (11)	C14—C13—H13A	110.2
O3—C12—C9	112.08 (10)	O3—C13—H13B	110.2
O3—C13—C14	107.33 (10)	C14—C13—H13B	110.2
C5—N1—H01	115.6 (11)	H13A—C13—H13B	108.5
C4—N1—H01	114.8 (11)	C13—C14—H14A	109.5
C5—N2—H02	109.4 (11)	C13—C14—H14B	109.5
C6—N2—H02	121.4 (11)	H14A—C14—H14B	109.5
C2—C1—H1A	109.5	C13—C14—H14C	109.5
C2—C1—H1B	109.5	H14A—C14—H14C	109.5
H1A—C1—H1B	109.5	H14B—C14—H14C	109.5
			
C1—C2—C3—C4	174.75 (13)	C7—C8—C9—C10	−1.97 (19)
C5—N1—C4—O1	−10.3 (2)	C7—C8—C9—C12	176.71 (11)
C5—N1—C4—C3	168.79 (12)	C8—C9—C10—C11	0.99 (19)
C2—C3—C4—O1	4.85 (19)	C12—C9—C10—C11	−177.65 (12)
C2—C3—C4—N1	−174.24 (12)	C9—C10—C11—C6	1.1 (2)
C6—N2—C5—N1	−174.22 (11)	C7—C6—C11—C10	−2.31 (19)
C6—N2—C5—S	4.13 (19)	N2—C6—C11—C10	−179.92 (12)
C4—N1—C5—N2	10.80 (19)	C13—O3—C12—O2	1.00 (18)
C4—N1—C5—S	−167.69 (10)	C13—O3—C12—C9	179.95 (10)
C5—N2—C6—C11	−42.44 (19)	C8—C9—C12—O2	7.60 (19)
C5—N2—C6—C7	139.90 (13)	C10—C9—C12—O2	−173.76 (13)
C11—C6—C7—C8	1.35 (19)	C8—C9—C12—O3	−171.36 (12)
N2—C6—C7—C8	179.06 (11)	C10—C9—C12—O3	7.28 (17)
C6—C7—C8—C9	0.81 (19)	C12—O3—C13—C14	−169.36 (11)

### Hydrogen-bond geometry (Å, °)

**Table:**

*D*—H···*A*	*D*—H	H···*A*	*D*···*A*	*D*—H···*A*
N2—H02···O1	0.82 (2)	1.92 (2)	2.653 (1)	148 (2)
N1—H01···O2^i^	0.79 (1)	2.20 (1)	2.957 (1)	160 (2)
C13—H13A···O1^ii^	0.99	2.58	3.363 (2)	136
C1—H1B···S^iii^	0.98	3.00	3.854 (2)	147
C13—H13B···S^iv^	0.99	2.96	3.577 (1)	122
C14—H14C···S^v^	0.98	2.98	3.821 (2)	144

Symmetry codes: (i) *x*−1, *y*−1, *z*; (ii) −*x*+1, −*y*+2, −*z*+1; (iii) *x*−1, *y*−1, *z*−1; (iv) *x*+1, *y*+1, *z*; (v) −*x*+2, −*y*+2, −*z*+2.
